# Specification of primordial germ cells in medaka (Oryzias latipes)

**DOI:** 10.1186/1471-213X-7-3

**Published:** 2007-01-11

**Authors:** Amaury Herpin, Stefan Rohr, Dietmar Riedel, Nils Kluever, Erez Raz, Manfred Schartl

**Affiliations:** 1 Department of Physiological Chemistry I, University of Wuerzburg, Biozentrum, Am Hubland, D-97074 Wuerzburg, Germany; 2 Department of Germ Cell Development, Max-Planck-Institute for Biophysical Chemistry, Am Fassberg 11, 37077 Göttingen, Germany; 3 Department of Neurobiology, Max-Planck-Institute for Biophysical Chemistry, Am Fassberg 11, 37077 Göttingen, Germany; 4 Germ Cell Development, Max-Planck-Institute for Biophysical Chemistry, Göttingen and Institute of Cell Biology, ZMBE, University of Münster, Germany; 5 Rudolf-Virchow Center for Experimental Medicine, University of Wuerzburg, Versbacher Straße 9, D-97074 Wuerzburg, Germany

## Abstract

**Background:**

Primordial germ cells (PGCs) give rise to gametes that are responsible for the development of a new organism in the next generation. Two modes of germ line specification have been described: the inheritance of asymmetrically-localized maternally provided cytoplasmic determinants and the induction of the PGC fate by other cell types.

PGCs specification in zebrafish appears to depend on inheritance of germ plasm in which several RNA molecules such as *vasa *and *nanos *reside. Whether the specification mode of PGCs found in zebrafish is general for other fish species was brought into question upon analysis of *olvas *expression – the *vasa *homologue in another teleost, medaka (*Oryzias latipes*). Here, in contrast to the findings in zebrafish, the PGCs are found in a predictable position relative to a somatic structure, the embryonic shield. This finding, coupled with the fact that *vasa *mRNA, which is localized to the germ plasm of zebrafish but does not label a similar structure in medaka opened the possibility of fundamentally different mechanisms governing PGC specification in these two fish species.

**Results:**

In this study we addressed the question concerning the mode of PGC specification in medaka using embryological experiments, analysis of RNA stability in the PGCs and electron microscopy observations. Dramatic alterations in the somatic environment, i.e. induction of a secondary axis or mesoderm formation alteration, did not affect the PGC number. Furthermore, the PGCs of medaka are capable of protecting specific RNA molecules from degradation and could therefore exhibit a specific mRNA expression pattern controlled by posttrancriptional mechanisms. Subsequent analysis of 4-cell stage medaka embryos using electron microscopy revealed germ plasm-like structures located at a region corresponding to that of zebrafish germ plasm.

**Conclusion:**

Taken together, these results are consistent with the idea that in medaka the inheritance of maternally provided asymmetrically-localized cytoplasmic determinants directs cells to assume the germ line fate similar to zebrafish PGCs.

## Background

Two basic strategies for the specification of primordial germ cells (PGCs) have been described. In *Drosophila*, *C. elegans *and *Xenopus *the inheritance of germ plasm with associated germ cell determinants appears to be required for PGC formation [1-3]. In these organisms, maternally provided asymmetrically-localized germ plasm, or germ plasm-like structures that are visible in electron micrographs (so called "nuage") are found in positions where the PGCs are formed. In *Drosophila *it has been shown that the germ plasm has an instructive role in directing nuclei to the germline [4] while zebrafish embryos from which the germ plasm was removed lacked germ cells [5]. In mouse (and by extension other mammals) and urodelean amphibians the mechanisms of germline specification are different. Here, morphologically distinct germ plasm has not been identified in early embryos but rather, as revealed in transplantation experiments, cellular interactions are thought to be responsible for PGC specification [6,7]. Consistently, the formation of the founding population of PGCs in the mouse was shown to depend on extracellular factors of the bone morphogenetic protein (BMP) family [8-11]. Whereas the actual specification and formation of PGCs in mammals and in urodeles appear to be independent of germ plasm, material resembling nuage, a germ plasm organelle, is found in germ cells of these organisms at later developmental stages [12,13].

Germ-cell specification in fish was studied mainly in bony fish (teleosts) where the work relied on observations using light and electron microscopy. The germ cells were recognized by their large size and by the typical electron dense nuage-like structures. Using these criteria, the earliest time point at which germ cells could be recognized in teleosts was around the onset of somitogenesis [14-16]. Some investigators deduced that the position where the PGCs were first identified was their site of origin (For references see [17]). Transplantation experiments of the embryonic shield, the fish organizer, in the teleost *Fundulus*, to the extraembryonic yolk sac of hosts resulted in, among other tissues, the development of germ cells and gonads with the highest frequency of germ cells found when the posterior quarter of the *Fundulus *shield was grafted [18,19]. One could not exclude the possibility however, that a small population of PGCs existed already before the end of gastrulation and migrated to the position where they were identified either morphologically or by embryological transplantation experiments. Thus, the question of the time and place of germ-cell origin in fish remained open.

**Figure 1:**
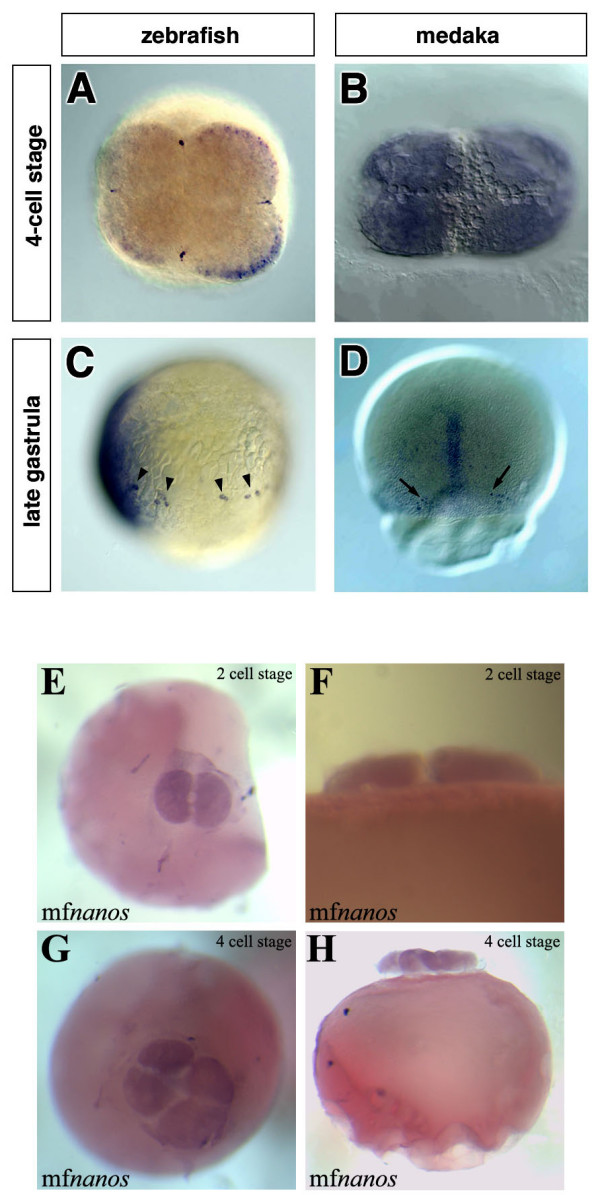
**The early expression patterns of zebrafish-vasa, medaka-olvas and medaka-nanos1 mRNAs**. In zebrafish, the *vasa *transcript is enriched at the marginal positions of the first two cleavage planes (A), while at a similar stage *olvas *mRNA is uniformly distributed throughout the cytoplasm of all blastomers (B). Throughout development in zebrafish, the *vasa *mRNA is expressed exclusively in the PGCs that can be found in random dorsoventral positions during blastula and gastrula stages (arrowheads in C). In contrast, at the first time point when medaka PGCs can be observed (stage 16), they are found on both sides of the embryonic shield on the dorsal side of the embryo (arrows in D). All images shown are whole-mount *in situ *hybridizations. The probes used are: *vasa *(A), *vasa *and *chordin *(C), *olvas *(B, D) and *nanos1 *(E to H). A, B, E and H are animal view, C is a lateral view with the dorsal aspect (labeled with *chordin*) to the left and D is a dorsal view, F and H are lateral views.

The first molecular marker for PGCs in fish is the zebrafish *vasa *mRNA [20] an ATP-dependent RNA helicase of the DEAD-box family [21,22] that is expressed in PGCs of many species (reviewed in [23]). *vasa *was originally identified in *Drosophila *as a maternal effect gene required for the formation of abdominal segments and for germ-cell specification [24]. Similar to many other organisms, zebrafish *vasa *mRNA is maternally supplied and it is detected in the PGCs during all stages of germ line development [20,25-28]. Close examination by whole-mount *in situ *hybridization at the 2- and 4-cell stages revealed that the *vasa *transcript is enriched at the marginal positions of the first two cleavage planes leading to the formation of four stripes of intense *vasa *RNA staining [20,25] (Fig. 1A). Interestingly, transmission electron microscopy showed that at the 4-cell stage *vasa *RNA is embedded within an electron dense matrix that resembles nuage, indicating that at this stage the RNA resides within the zebrafish germ plasm [28]. The position of the germ plasm and its early segregation among the first blastomeres strongly support the notion that in zebrafish cells are directed to the germ line fate by the action of determinants present in the germ plasm [28]. Consistently, inhibiting the translation of RNA molecules that are localized to the germ plasm of the zebrafish can result in severe defects in PGC development (e.g. [29,30]).

The findings obtained in zebrafish were extended to suggest that generally in fish, PGCs are specified by determinants found in the germ plasm and that the position of the germ plasm could be inferred from the position of RNA molecules such as *vasa *and *nanos *which mark the early germ plasm. Surprisingly, the cloning of the *vasa *and *nanos *genes from another teleost, medaka (*Oryzias latipes*) and analysis of their mRNA expression patterns did not provide direct support for the notion that germ plasm is responsible for PGC specification in all fish species [31,32]. In contrast to the enrichment of *vasa *transcripts in the position of the germ plasm observed in zebrafish, the transcripts of *olvas*, the medaka *vasa *homologue, were uniformly distributed until stage 16 at late gastrulation ([31], Fig. 1B and 1D). At this stage, PGCs, recognized by higher levels of *olvas *expression, are found on either side of the shield, the fish organizer [31]. Similarly, medaka *nanos *transcripts did not aggregate at the two first cleavage planes (Fig. 1E to 1H). As mentioned above, in zebrafish the germ plasm contains *vasa *and *nanos *mRNAs and is found at the distal parts of the first two cleavage planes. These four locations are randomly oriented relative to the dorsal aspect of the embryo leading to specification of PGCs in random dorsoventral positions [26]. In contrast, at these stages, *olvas *and *nanos *mRNAs were found to be uniformly distributed in the corresponding stages and when the PGCs are first observed using *olvas *as a molecular marker, they are found in a defined position relative to somatic structures. If the early expression site of *vasa *and its homologues indeed mark the site and stage of PGC origin, then the expression pattern of *olvas *would imply a different mechanism for specifying PGC in medaka.

In this work we investigate the question of the mechanisms responsible for PGC specification in medaka. We determine the effect of perturbations in somatic development on PGC specification and describe the properties of medaka PGCs regarding the metabolism of specific RNA molecules. The results presented here are consistent with the notion that despite the differences in the expression pattern of *vasa *during early embryogenesis in these two teleosts that represent totally different branches of the phylogenetic tree, it is likely that similar basic mechanisms are responsible for PGC specification.

## Results and Discussion

Several lines of evidence strongly indicate that in mouse the PGC fate is induced by signals that originate in adjacent tissues. In this organism, germ cells arise just before or during early gastrulation, around the proximal part of the epiblast adjacent to extra-embryonic tissues [6-11]. The finding that medaka PGCs are first detected at a specific position relative to the embryonic shield ([31] and Fig. 1D) could be interpreted as evidence for induction of PGCs by the adjacent somatic cells. Consistent with this suggestion, axial mesodermal tissues in fish are known to be capable of inducing cell fate in adjacent tissues (e.g. [33]).

### Medaka PGCs number is independent of patterning of somatic tissues

**Figure 2:**
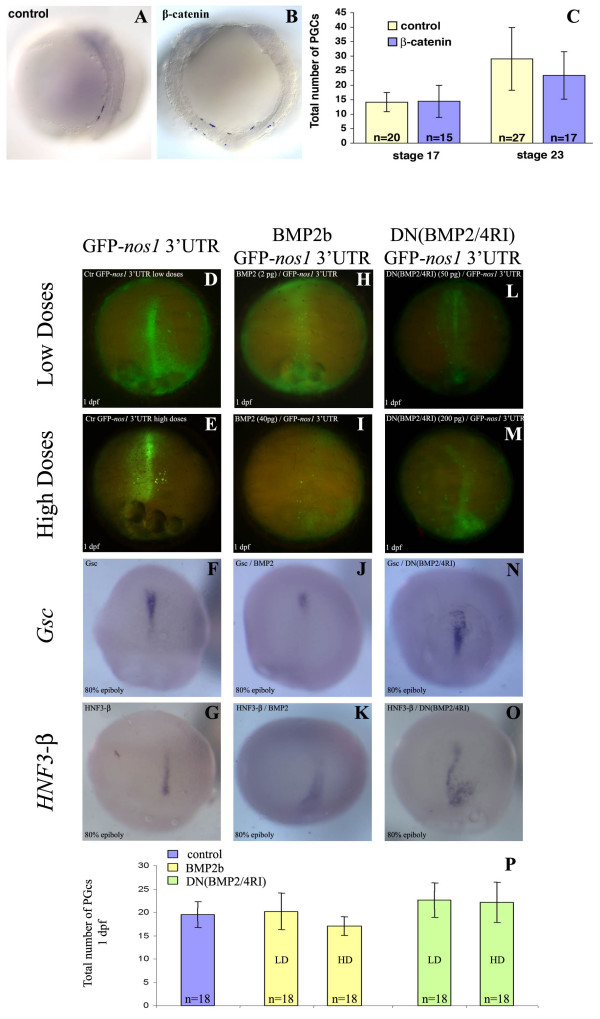
**The effect of axis duplication and mesoderm induction modulation in medaka on PGC number**. (A, B and C) Induction of a complete secondary embryonic axis was achieved by injection of β-*catenin *at the 8- and 16-cell stage. The injected embryos were fixed at stages 17 and 23 and their PGCs visualized by *in situ *hybridization using an *olvas *antisense RNA probe. A: a stage 23 control embryo injected with GFP-*globin*; B: a stage 23 embryo with a duplicated embryonic axis. In both stages analyzed, the average PGC number in embryos with a duplicated axis is similar to that of control embryos (C). (D to P) BMP-mediated mesoderm induction modulation was achieved by injection of either BMP2 (H to K) or dominant negative form of BMP2/4RI (DN(BMP2/4RI)) (L to O). To confirm mesoderm formation alteration, *in situ *hybridization was performed with either *Goosecoid *(*Gsc*) ventrolateral mesodermal marker (F, J and N) or *HNF3-b *axial mesodermal marker (G, K and O). One day post fertilization (dpf), the average PGC number in embryos with altered mesoderm is similar to that of control embryos (P). The number of embryos analyzed is provided within the corresponding bars.

If PGCs in medaka are induced through interactions with somatic tissues at the site where they are first observed, then specific alterations in the development of these tissues are expected to bring about changes in the position and number of PGCs. To address this point early somatic development was affected in two ways: either by injecting b-catenin thereby generating two embryonic axes in a single embryo (Fig. 2A,B and 2C), or by disturbing early axial mesoderm specification through BMP pathway modulation (Fig. 2D to 2P).

The first manipulation was carried out by injection of 600 pg β-*catenin *RNA into one marginal blastomere at the 8- or 16-cell stage (based on the protocol for zebrafish by [34]). This resulted in formation of a complete secondary axis in about 3% of the injected embryos. Control embryos were injected with the same amount of GFP-*globin *RNA. The injected embryos were fixed at stages 17 and 23 and whole-mount *in situ *hybridization was performed using an *olvas *antisense RNA probe to visualize the PGCs (for stage 23 see Fig. 2A and 2B). The position of PGCs in embryos with an induced secondary embryonic axis was similar to that observed in control embryos of the same developmental stage. If PGC fates are indeed induced by somatic tissues of the organizer, one would expect an increase in PGC number when two shields are present in the embryo. However, the average number of PGCs in embryos with duplicated axes was found to be similar to the number of PGCs in control embryos (Fig. 2C).

Mesoderm patterning during early gastrulation was modulated by either overexpressing BMP2 (Fig. 2H to 2K) or expressing a dominant negative form of BMP2/4 receptor I (DN(BMP2/4RI)) (Fig. 2L to 2O), which results in ventralization and dorsalization of mesoderm, respectively. Again, no alteration in the total number of PGCs was observed, although their localization was severely impaired (Fig. 2D to 2P). Thus, the number of PGCs is independent of mesoderm patterning.

These findings do not support the idea that PGC fate is induced by neighboring cells at the site where they are first detected. Rather, these results are more consistent with the model according to which PGCs in medaka are specified by inheritance of pre-localized maternal components whose amount is the limiting factor determining the number of PGCs. As BMP-induced mesoderm formation occurs during gastrulation and the induction of the secondary axis by β-*catenin *arises at a very early stage of development well before gastrulation initiates, we consider it unlikely that the PGCs are specified by an earlier inductive process.

### Medaka PGCs selectively stabilize maternally provided transcripts

As mentioned above, in *Drosophila *and *C. elegans *the PGCs are specified by inheritance of maternally provided asymmetrically-localized cytoplasmic determinants. In these organisms, maternally provided transcripts that are expressed in the PGCs are selectively stabilized within these cells while being degraded in somatic cells [35,36]. Similarly, in zebrafish the mRNA of the *vasa *gene [37], as well as *nanos*1 (*nos*1) mRNA [29,38] are degraded in somatic cells but are stabilized and translated in cells that contain germ plasm.

If specification of PGCs in medaka occurs by inheritance of germ plasm similar to zebrafish, then the phenomena of differential stability of RNAs between somatic and primordial germ cells is expected to take place in this species, as well.

**Figure 3:**
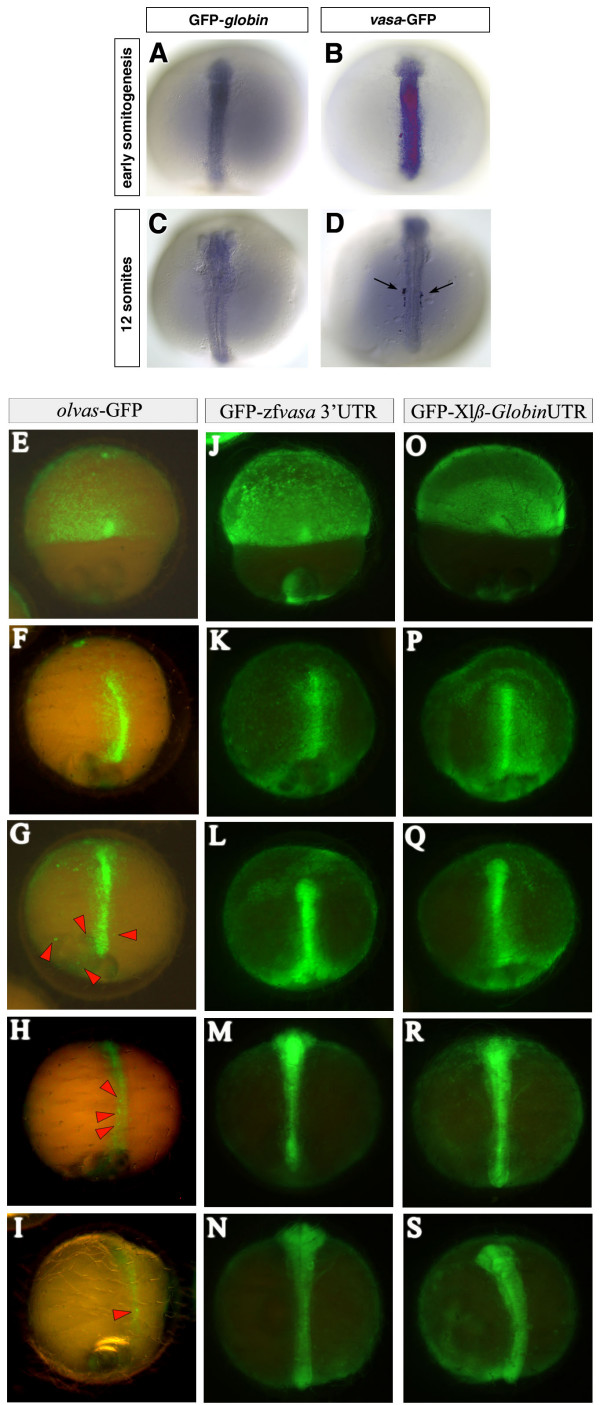
**Stabilization of zebrafish vasa and translational regulation of medaka olvas mRNA in PGCs of medaka**. 100 pg of GFP tagged fusion RNAs were injected at the 2-cell stage. (A to D) The embryos were fixed at early somitogenesis and at 12-somite stage and *in situ *hybridization using a GFP antisense RNA probe was performed to reveal the spatial distribution of the injected RNAs. In the control embryos the GFP-*globin *RNA is degraded uniformly in all cells (A and C) whereas in *vasa*-GFP injected embryos (B and D), the PGCs retain the injected RNA (arrows in D) while it is degrading in somatic cells. (E to S) *olvas*-GFP (E to I) as well as GFP-zf*vasa *3'UTR (J to N) constructs were injected in medaka and followed for GFP expression and compared to control GFP-*β globin *3'UTR injected embryos (O to S). Interestingly, in medaka, *olvas*-GFP protein expression was rapidly restricted to PGCs (E to I), while zebrafish *vasa *3'UTR did not induced selective GFP expression.

To examine this point, mRNAs from three zebrafish GFP-fusion constructs, *vasa*-GFP, GFP-zf*vasa *3'UTR and GFP-*nos*1 3'UTR, were injected into 1- to 2-cell stage medaka embryos. Following the injection of 100 pg RNA, *in situ *hybridization using an antisense GFP-RNA probe was performed at different times of development to reveal the spatial distribution of the injected fusion RNAs. As a control, RNA encoding GFP flanked by the *Xenopus laevis*-*globin *5' and 3'UTRs was injected and the distribution of this RNA was similarly followed at different stages of development. Remarkably, the zebrafish *vasa*-GFP-fusion RNA undergoes rapid degradation in the soma and is stabilized in the germ line cells (arrows in Fig. 3D). An analogous distribution is observed for GFP-*nos*1 3'UTR RNA injected into medaka embryos (data not shown). Interestingly, the results resemble those obtained in zebrafish in several ways: 1. These injected GFP-tagged RNA molecules do not aggregate at the cleavage planes (data not shown). 2. The injected RNA molecules exhibit differential stability between somatic and primordial germ cells. Whereas at the onset of somitogenesis the soma is stained strongly with the antisense GFP probe (Fig. 3B), at stage 23 (12 somites) virtually only the PGCs retain the injected *vasa*-GFP-fusion RNA (Fig. 3D). In contrast, control embryos show no specific stabilization of the GFP-*globin *RNA in the germ line but instead, exhibit general RNA degradation in all cells (Fig. 3A and 3C). Consistent with the notion that as in zebrafish, post transcriptional events control differential gene expression between germ cells and somatic cells in other fish species as well, uniform introduction (by injection) of GFP- *nos1 *3'UTR RNA into 1-cell embryos of *Oryzias latipes *[see Additional file 2], *O. curvinotus *[see Additional file 3], *O. luzonensis *[see Additional file 4] or *Betta splendens *[see Additional file 5] resulted in germ cells specific GFP expression [see Additional file 1]. Therefore, differential (*nos1 *3'UTR)-induced post-transcriptional events between somatic and germ cells appears to be a mechanism conserved in different fish lineages representing distantly related branches of the phylogenetic tree (Cypriniformes, Perciformes and Beloniformes).

To determine whether this mechanism is relevant for the observed *olvas *mRNA and protein distributions, a reciprocal experiment was performed in which the original full-length *olvas *RNA was injected in zebrafish embryos. The analysis of its fate with an antisense *olvas *probe revealed no specific stabilization of *olvas *RNA in the PGCs of zebrafish (data not shown). Thus, neither medaka *olvas *3'UTR nor *olvas *open reading frame (ORF) mediate germ cell specific RNA stability in zebrafish. Interestingly, in medaka, *olvas*-GFP protein expression was rapidly restricted to PGCs (Fig. 3E to 3I), although the *olvas*-GFP fusion RNA itself was not efficiently differentially stabilized in the PGCs of medaka (data not shown). Therefore, the preferential localization of the *olvas*-GFP protein to the PGCs (Fig. 3E to 3I) could be attributed to complementary specific posttranscriptional mechanisms: (i) in agreement with Kurokawa *et al*., a PGC-specific enhanced translation due to the *olvas *3'UTR [32]. (ii) Somatic inhibition of translation due to the open reading frame itself. (iii) Soma-specific destabilization of the protein [37].

In addition, like *olvas *3'UTR in medaka, zebrafish *vasa *3'UTR has been shown to be essential for proper protein PGC localization in zebrafish. Hence, when injected in medaka, GFP-zf*vasa *3'UTR failure of selective GFP expression (Fig. 3J to 3N compared to Fig. 3E to 3I and 3O to 3S) points out possible specie-variability of either targeted cis-acting RNA sequence elements or RNA-interacting protein sequence, but likely using a similar conserved mechanism.

Thus, while we provide evidence for the existence of conserved mechanisms governing PGCs specification in zebrafish and medaka, the similarities apparently can not be extended to the control of PGC-specific expression of every molecule, as shown here for the zf-*vasa *and *olvas *RNAs. Hence, the mechanisms directing PGC-specific expression of *olvas *could depend on combined machineries associating enhanced/repressed specific soma/germline translation, protein stabilization and possibly, to a lower extend, RNA stability.

### Identification of germ plasm like structures in early Medaka embryos

**Figure 4:**
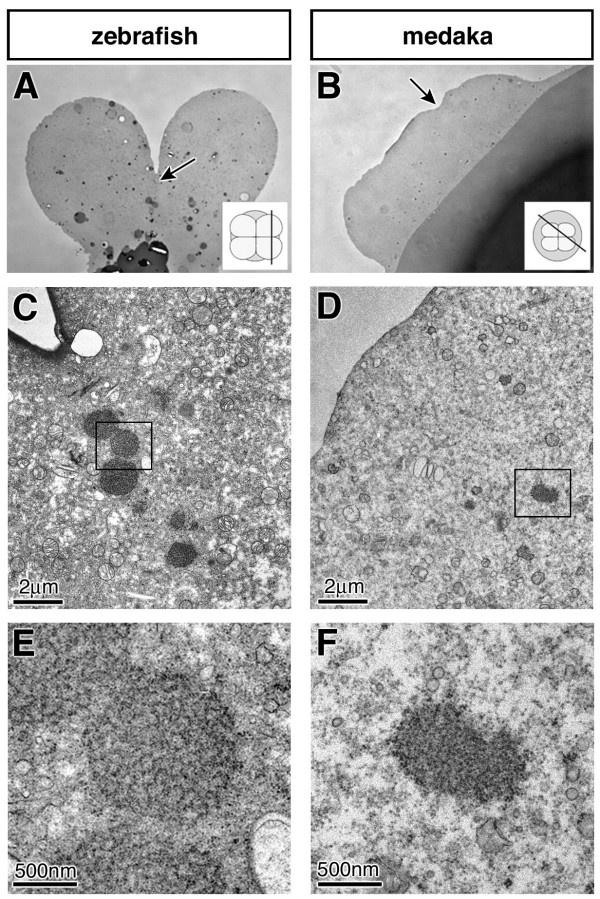
**Sections of early 4-cell stage zebrafish and medaka embryos**. Semi-thin section of zebrafish (A) and medaka (B) embryos visualized in a light microscope. Arrows indicate the areas shown in C-F representing the cleavage positions. The insets represent the orientation of the sections. Low magnification at the electron microscope showing the region of the germ plasm of zebrafish (C), and the corresponding position in medaka (D). At a higher magnification, the germ plasm of zebrafish is visible as distinct amorphous inclusions with a regular spherical shape and a filamentous fine structure lacking a surrounding membrane (E). At this magnification germ plasm resembling structure composed of irregularly shaped amorphous inclusions with a granular fine structure can be observed in medaka embryos. As described for zebrafish [28], the putative germ plasm of medaka can be followed in serial sections.

As the results presented above are consistent with the idea that in medaka, like in zebrafish, inheritance of germ plasm is responsible for the formation of PGCs, we sought to identify germ plasm in early medaka embryos. Both in birds and fish the detection of germ plasm in early embryos was possible only after molecular markers for this organelle became available (the Vasa protein and mRNA respectively) [28,39]. In the absence of such a molecular marker for the medaka germ plasm we concentrated our efforts on positions equivalent to those where zebrafish germ plasm is found. In zebrafish, the germ plasm with its characteristic electron dense appearance was found to be concentrated below the first two cleavage furrows in 4-cell stage embryos [28]. In medaka, the first 4 blastomeres are much smaller in size and the cleavage furrows are not as pronounced as they are in zebrafish (compare Fig. 4A and 4B). However, within the cytoplasm at the areas where the 4 cells are in contact, distinct electron-dense structures were identified. These structures are formed of amorphous inclusions with a granular fine structure, lacking a surrounding membrane (Fig. 4D and 4F). Serial sections show that this is a rod-like structure approximately 0.5 μm in diameter. These features of the structure correspond to descriptions of germ plasm in many species [40]. The fact that this structure is found exclusively beneath the cleavage furrows, similar to the germ plasm in zebrafish at the corresponding stage leads us to suggest that the observed electron-dense material represents the germ plasm of medaka.

## Conclusion

Our findings in medaka are consistent with the suggestion that the mode of PGC specification is conserved with zebrafish and possibly other teleosts. First, we show that dramatic alterations in somatic development (axis duplication and mesoderm formation alteration) do not alter the PGC number in medaka, which appears inconsistent with an inductive mode of PGC specification that depend on signals from somatic cells. Second, the process of germ-line specific RNA stabilization shown here for medaka has been described for those organisms whose PGCs are specified by inheritance of maternally provided cytoplasmic determinants. Consistently, material resembling germ plasm was identified in 4-cell stage medaka embryos. The apparent paradox that the *vasa*-homologue of medaka differs from its zebrafish counterpart with respect to its early spatial distribution and its ability to be subjected to the "degradation-protection" control mechanism may be reconciled by the finding that *vasa *mRNA appears to be dispensable for early PGC development in zebrafish [41].

## Methods

### Synthesis of RNA for Embryo Injections

β-*catenin *sense RNA was transcribed from pT_7_Ts [34], which contains the full-length zebrafish β-*catenin *cDNA. The injection of the zebrafish 600 pg β-*catenin *RNA into one of the marginal blastomeres of 8- and 16-cell stage medaka embryos induced complete secondary axis in 3% of the embryos. As a control, embryos were injected with 600 pg of GFP-*globin *RNA.

Zebrafish BMP2b and dominant negative truncated version of BMP2/4 receptor I (DN(BMP2/4RI)) were transcribed from pSP64TS and pSP64T respectively. Capped synthetic mRNA were injected (1 nL) into the cytoplasm of a single-cell Medaka embryo at different concentrations (2 to 50 ng/μL and 40 to 200 ng/μL for BMP2b and DN(BMP2/4RI) respectively).

The *vasa*-GFP construct includes the zebrafish full-length *vasa *cDNA into which the mmGFP5 [42] open reading frame (ORF) was inserted [37].

The GFP-*nos*1 3'UTR construct includes the mmGFP5 ORF cloned upstream of the 3'UTR of the zebrafish *nanos*1 gene [29]. The GFP-zf*vasa *3'UTR construct was made by inserting the PCR amplified zebrafish *vasa *3'UTR after GFP (*Xho*I crated site).

The *olvas*-GFP construct was prepared by inserting the mmGFP5 ORF into the *Sac*II site in the full-length *olvas *cDNA (obtained from the *cab*-wild-type strain using the forward oligo AAAAAGCTTTCAGTTTGAAGCTAACAGCAGCAC and reverse oligo AAATCTAGATTTGKTGAAAACTTTTAATTATYAGGAGAA.

GFP-*globin*: mmGFP5 was cloned in between the *globin *5'UTR and *globin *3'UTR of pSP64T [37].

All RNA molecules were synthesized using the mMessage mMachinekit (Ambion).

### *In situ* Hybridization

RNA whole-mount *in situ *hybridization using *olvas *[31], *nanos *[32]*goosecoid (Gsc) *or *HNF3-b *DIG labeled probes were performed as previously described for zebrafish [26].

### Electron Microscopy

For transmission electron microscopy individual eggs were fixed by immersion in a solution containing 4% paraformaldehyde and 0.1% glutaraldehyde in 0.1 M phosphate buffer at pH 7.4 for 4 h. The chorion was subsequently removed and the eggs were fixed further overnight at 4°C using 2% glutaraldeahyde in 0.1 M phosphate buffer at pH 7.4. After an additional fixation with 1% OsO_4 _and pre-embedding staining with 1% uranyl acetate, eggs were dehydrated and embedded in EMbed 812 resin (Plano).

The sectioning was performed using a Leica Ultracut ultramicrotome. 600 nm thick sections were stained with tolouidine blue and visualized in a Zeiss light microscope to select the area of interest. Thereafter, 60 nm thick sections were collected and counterstained with 1% uranyl acetate and lead citrate and examined using a Philips CM 120 BioTwin (Philips Inc.Eindhoven, The Netherlands) transmission electron microscope.

### Fish Maintenance and Staging

Medaka (*Oryzias latipes*) wild-type strain *cab *was maintained as described previously for zebrafish [43]. Developmental stages were determined according the criteria set out by [44].

## Authors' contributions

ER and MS conceived the study. AH performed experiments for Fig. 1E to 1H, 2D to 2P, 3E to 3S, Additional files 1, 2 and 3 to 5; SR for Fig. 1A to 1D, 2A to 2C and 3A to 3D; DR for Fig. 4; NK for Fig. 1E to 1H. SR and AH wrote the first draft of the manuscript and all authors contributed to the writing of its final version. All authors read and approved the final manuscript.

## Supplementary Material

Additional File 1 **Zebrafish nanos1 3'UTR drives PGC-specific GFP/RFP expression**. To confirm Zebrafish *nanos1 *3'UTR driven PGC-specific expression, the RFP- Zf*nos1 *3'UTR construct was injected in the Medaka Olvas transgenic strain (A) expressing GFP under the control of the vasa-promoter [45]. Overlap of GFP (B) and RFP (C) indeed confirm PGC specific expression under the control of Zebrafish *nanos1 *3'UTR. Click here for file

Additional File 2 **PGC migration in medaka**. Time-lapse imaging of normal PGC migration during early development of GFP-*nos1 *3' UTR injected Medaka (*Oryzias latipes*) single embryo. Click here for file

Additional File 3 **Evidences for conserved nanos1 3' UTR-driven mechanisms governing teleost PGC-specific expression**. GFP-*nos1 *3' UTR capped RNA were injected at one cell stage in embryos of *Oryzias curvinotus *and GFP expression monitored for the same injected embryo. Primordial germ cell GFP-specific expression was observed. Click here for file

Additional File 4 **Evidences for conserved nanos1 3' UTR-driven mechanisms governing teleost PGC-specific expression**. GFP-*nos1 *3' UTR capped RNA were injected at one cell stage in embryos of *Oryzias luzonensis *and GFP expression monitored for the same injected embryo. Primordial germ cell GFP-specific expression was observed. Click here for file

Additional File 5 **Evidences for conserved nanos1 3' UTR-driven mechanisms governing teleost PGC-specific expression**. GFP-*nos1 *3' UTR capped RNA were injected at one cell stage in embryos of *Betta splendens *and GFP expression monitored for the same injected embryo. Primordial germ cell GFP-specific expression was observed. Click here for file
