# Supplementary figures and images for: Specification of primordial germ cells in medaka (Oryzias latipes)
Source: BMC Dev Biol. 2007 Jan 11;7:3. doi: 10.1186/1471-213X-7-3 (PMC1781431; doi:10.1186/1471-213X-7-3)

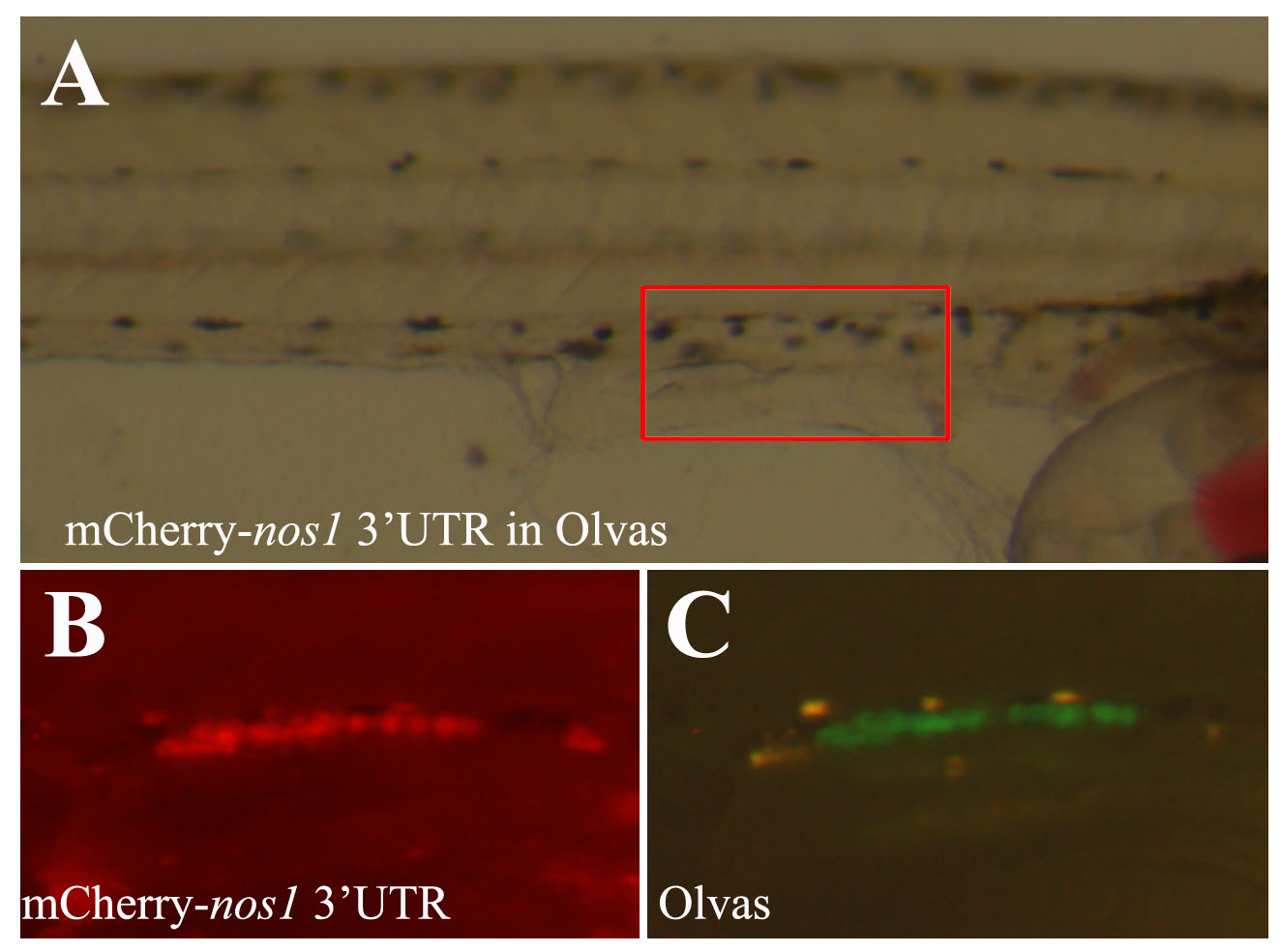

Supplement: Additional File 1 — Zebrafish nanos1 3'UTR drives PGC-specific GFP/RFP expression. To confirm Zebrafish nanos1 3'UTR driven PGC-specific expression, the RFP- Zfnos1 3'UTR construct was injected in the Medaka Olvas transgenic strain (A) expressing GFP under the control of the vasa-promoter [45]. Overlap of GFP (B) and RFP (C) indeed confirm PGC specific expression under the control of Zebrafish nanos1 3'UTR. [file 1471-213X-7-3-S1.jpeg]

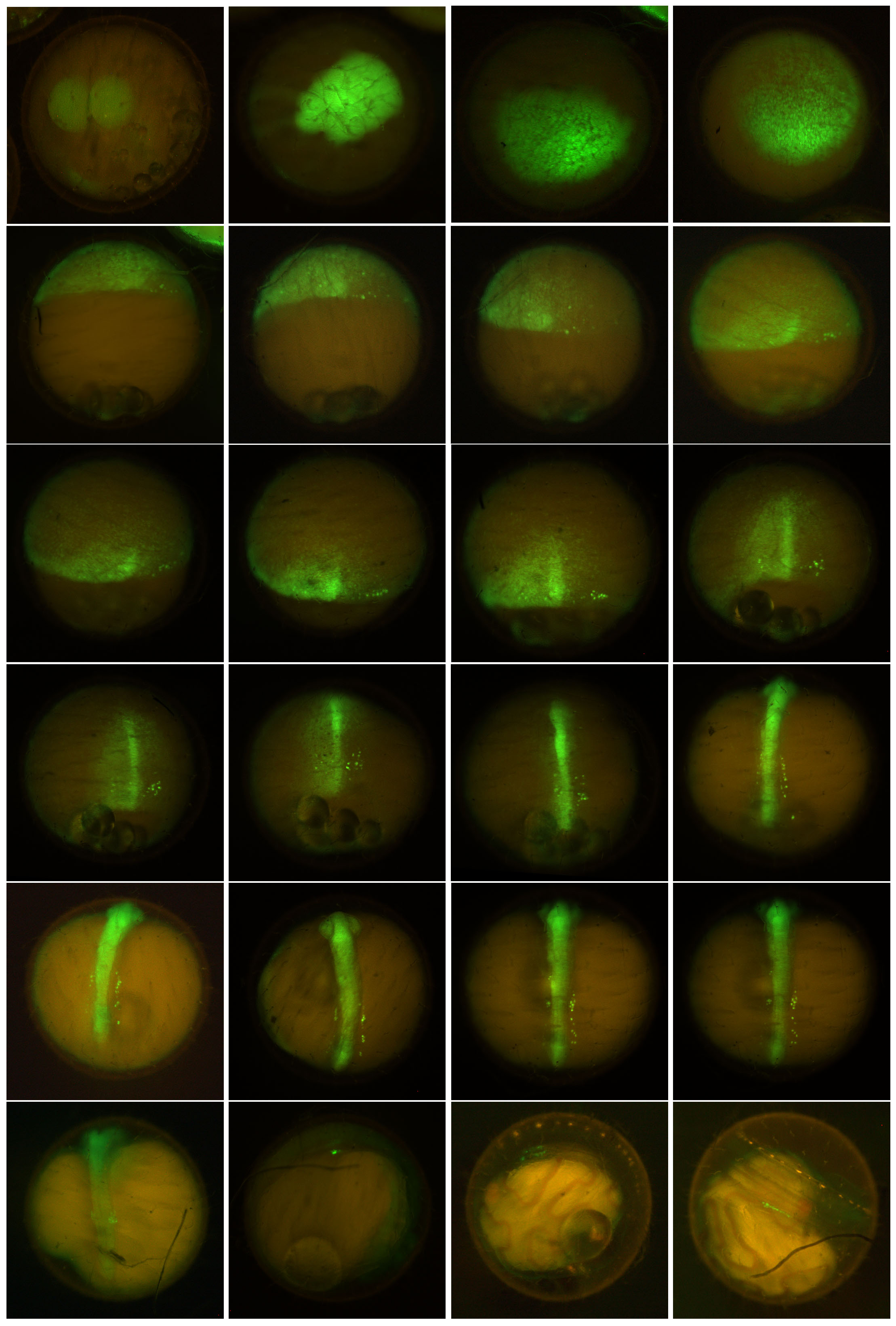

Supplement: Additional File 2 — PGC migration in medaka. Time-lapse imaging of normal PGC migration during early development of GFP-nos1 3' UTR injected Medaka (Oryzias latipes) single embryo. [file 1471-213X-7-3-S2.jpeg]

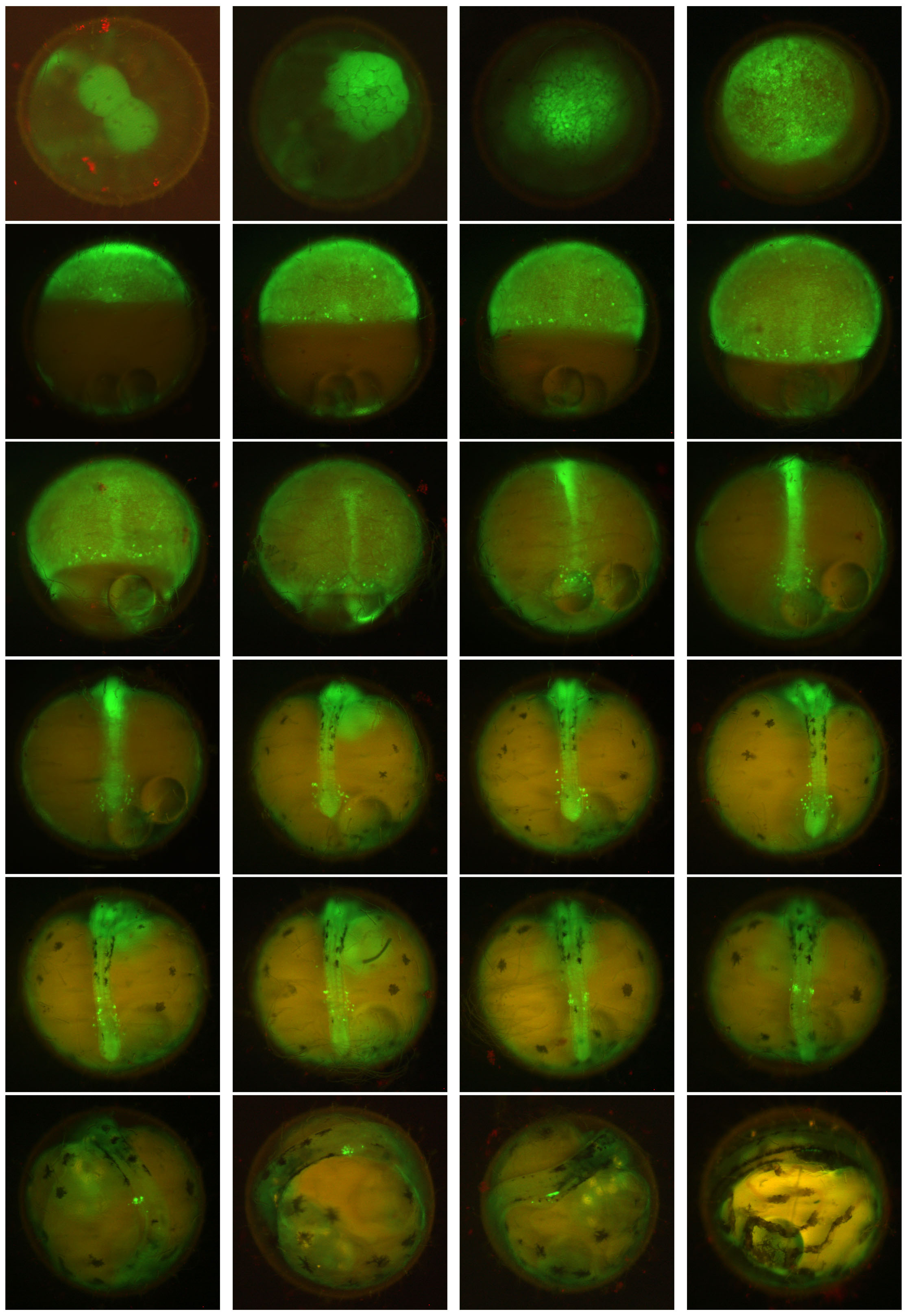

Supplement: Additional File 3 — Evidences for conserved nanos1 3' UTR-driven mechanisms governing teleost PGC-specific expression. GFP-nos1 3' UTR capped RNA were injected at one cell stage in embryos of Oryzias curvinotus and GFP expression monitored for the same injected embryo. Primordial germ cell GFP-specific expression was observed. [file 1471-213X-7-3-S3.jpeg]

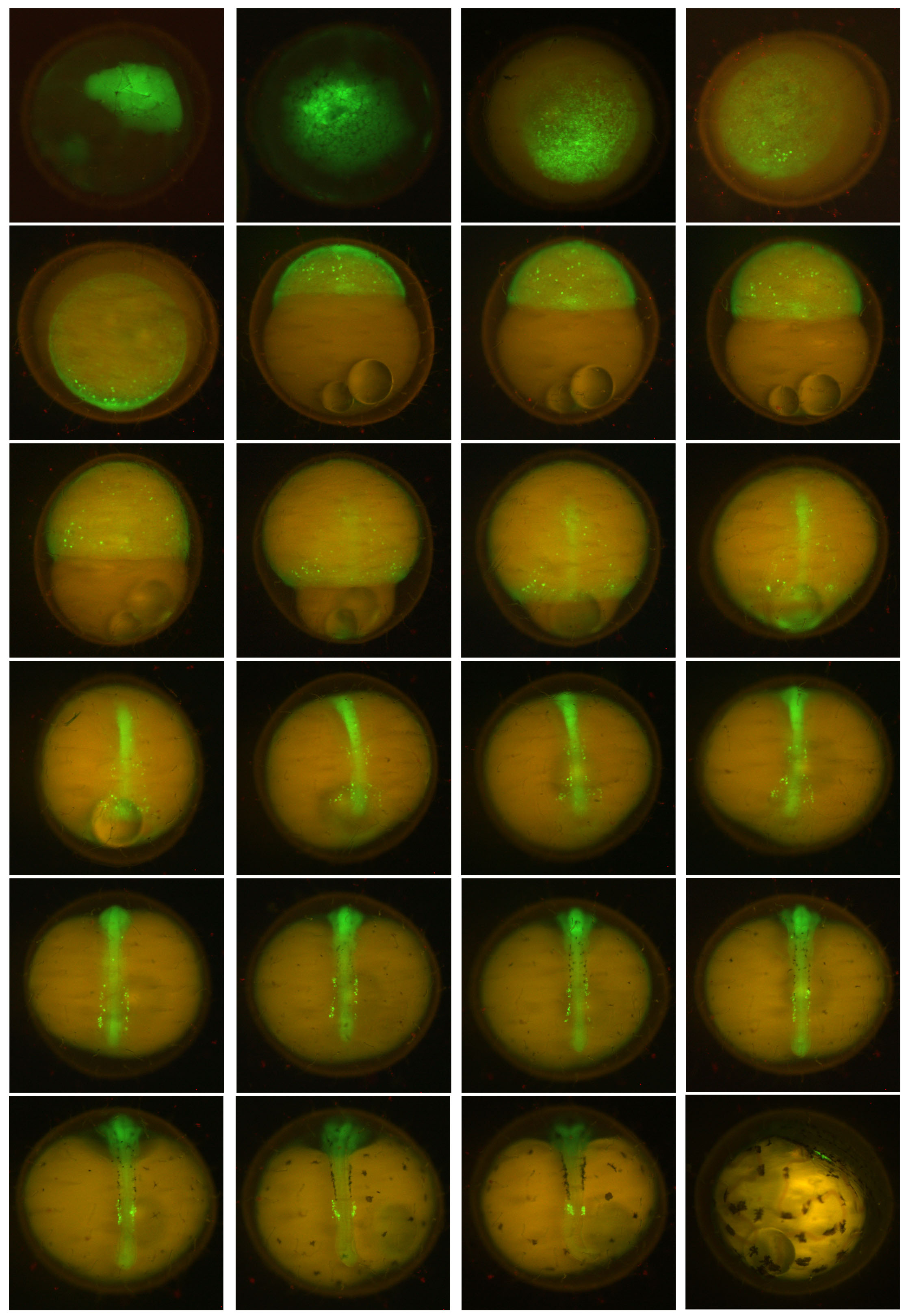

Supplement: Additional File 4 — Evidences for conserved nanos1 3' UTR-driven mechanisms governing teleost PGC-specific expression. GFP-nos1 3' UTR capped RNA were injected at one cell stage in embryos of Oryzias luzonensis and GFP expression monitored for the same injected embryo. Primordial germ cell GFP-specific expression was observed. [file 1471-213X-7-3-S4.jpeg]

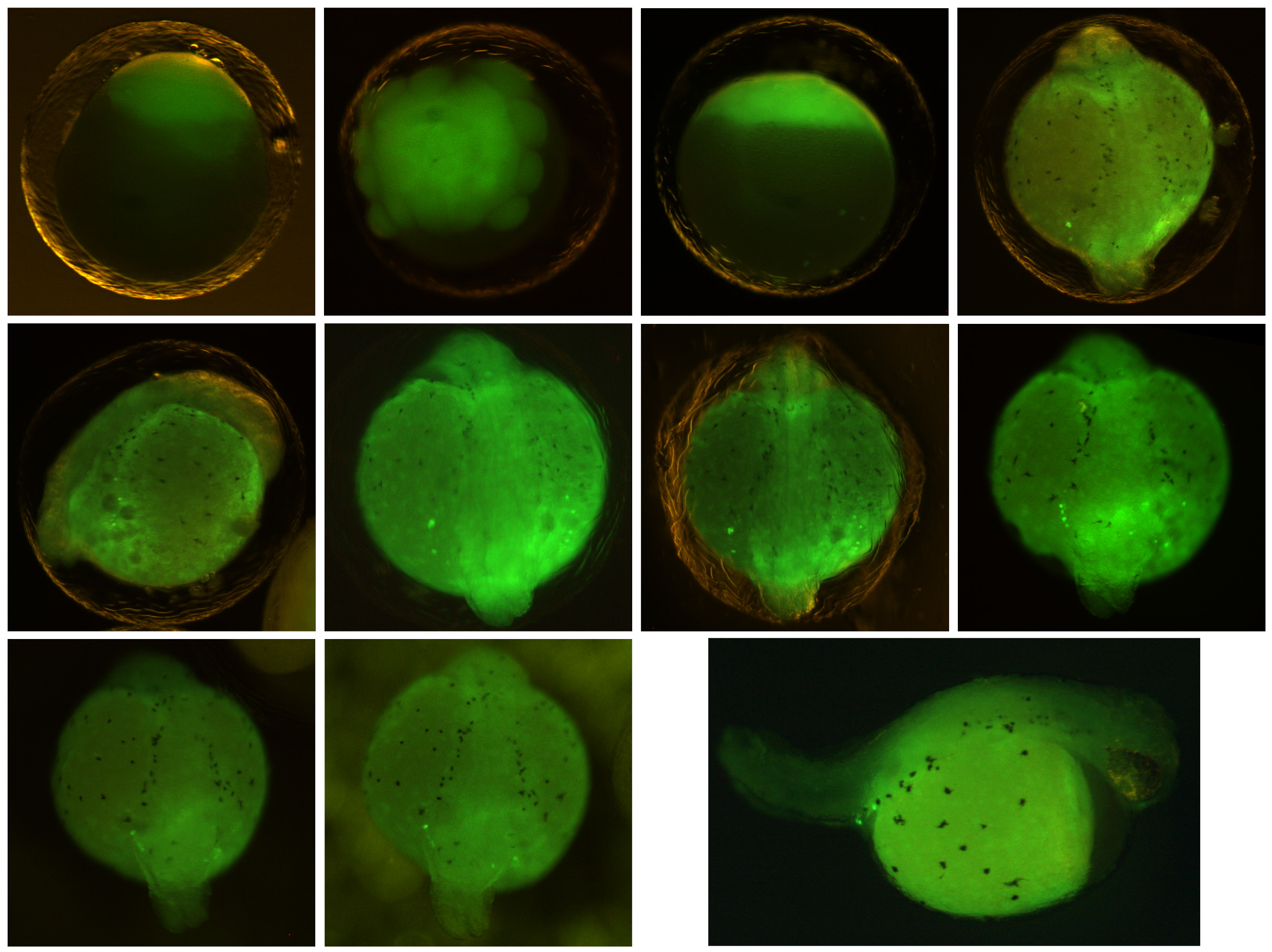

Supplement: Additional File 5 — Evidences for conserved nanos1 3' UTR-driven mechanisms governing teleost PGC-specific expression. GFP-nos1 3' UTR capped RNA were injected at one cell stage in embryos of Betta splendens and GFP expression monitored for the same injected embryo. Primordial germ cell GFP-specific expression was observed. [file 1471-213X-7-3-S5.jpeg]
